# Strength and Regulation of Seven rRNA Promoters in *Escherichia coli*

**DOI:** 10.1371/journal.pone.0144697

**Published:** 2015-12-30

**Authors:** Michihisa Maeda, Tomohiro Shimada, Akira Ishihama

**Affiliations:** 1 Meiji University, Faculty of Agriculture Chemistry, Kawasaki, Kanagawa 214–8571, Japan; 2 Chemical Resources Laboratory, Tokyo Institute of Technology, Nagatsuda, Yokohama 226–8503, Japan; 3 Research Center for Micro-Nano Technology, Hosei University, Koganei, Tokyo 184–8584, Japan; Niels Bohr Institute, DENMARK

## Abstract

The model prokaryote *Escherichia coli* contains seven copies of the rRNA operon in the genome. The presence of multiple rRNA operons is an advantage for increasing the level of ribosome, the key apparatus of translation, in response to environmental conditions. The complete sequence of *E*. *coli* genome, however, indicated the micro heterogeneity between seven rRNA operons, raising the possibility in functional heterogeneity and/or differential mode of expression. The aim of this research is to determine the strength and regulation of the promoter of each rRNA operon in *E*. *coli*. For this purpose, we used the double-fluorescent protein reporter pBRP system that was developed for accurate and precise determination of the promoter strength of protein-coding genes. For application of this promoter assay vector for measurement of the rRNA operon promoters devoid of the signal for translation, a synthetic SD sequence was added at the initiation codon of the reporter GFP gene, and then approximately 500 bp-sequence upstream each 16S rRNA was inserted in front of this SD sequence. Using this modified pGRS system, the promoter activity of each *rrn* operon was determined by measuring the *rrn* promoter-directed GFP and the reference promoter-directed RFP fluorescence, both encoded by a single and the same vector. Results indicated that: the promoter activity was the highest for the *rrnE* promoter under all growth conditions analyzed, including different growth phases of wild-type *E*. *coli* grown in various media; but the promoter strength of other six *rrn* promoters was various depending on the culture conditions. These findings altogether indicate that seven rRNA operons are different with respect to the regulation mode of expression, conferring an advantage to *E*. *coli* through a more fine-tuned control of ribosome formation in a wide range of environmental situations. Possible difference in the functional role of each rRNA operon is also discussed.

## Introduction

Bacteria carry a sophisticated genetic system to optimize the cell growth rate in response to environmental conditions. Growth rate is closely related to the level of ribosome synthesis, the key apparatus of protein synthesis. The number of ribosomes per cell in a growing bacterial cell increases in direct proportion to the growth rate [1,2]. The number of ribosomes per cell is proportional to the growth rate to satisfy the cells demand for protein synthesis [3]. In fast growing *E*. *coli* cells, there are as many as 70,000 ribosomes per cell, while at lower growth rates, this number is reduced to less than 20,000 [4]. The intracellular level of RNA polymerase, the key apparatus of transcription, correlates the ribosome level [5,6].

**Fig 1:**
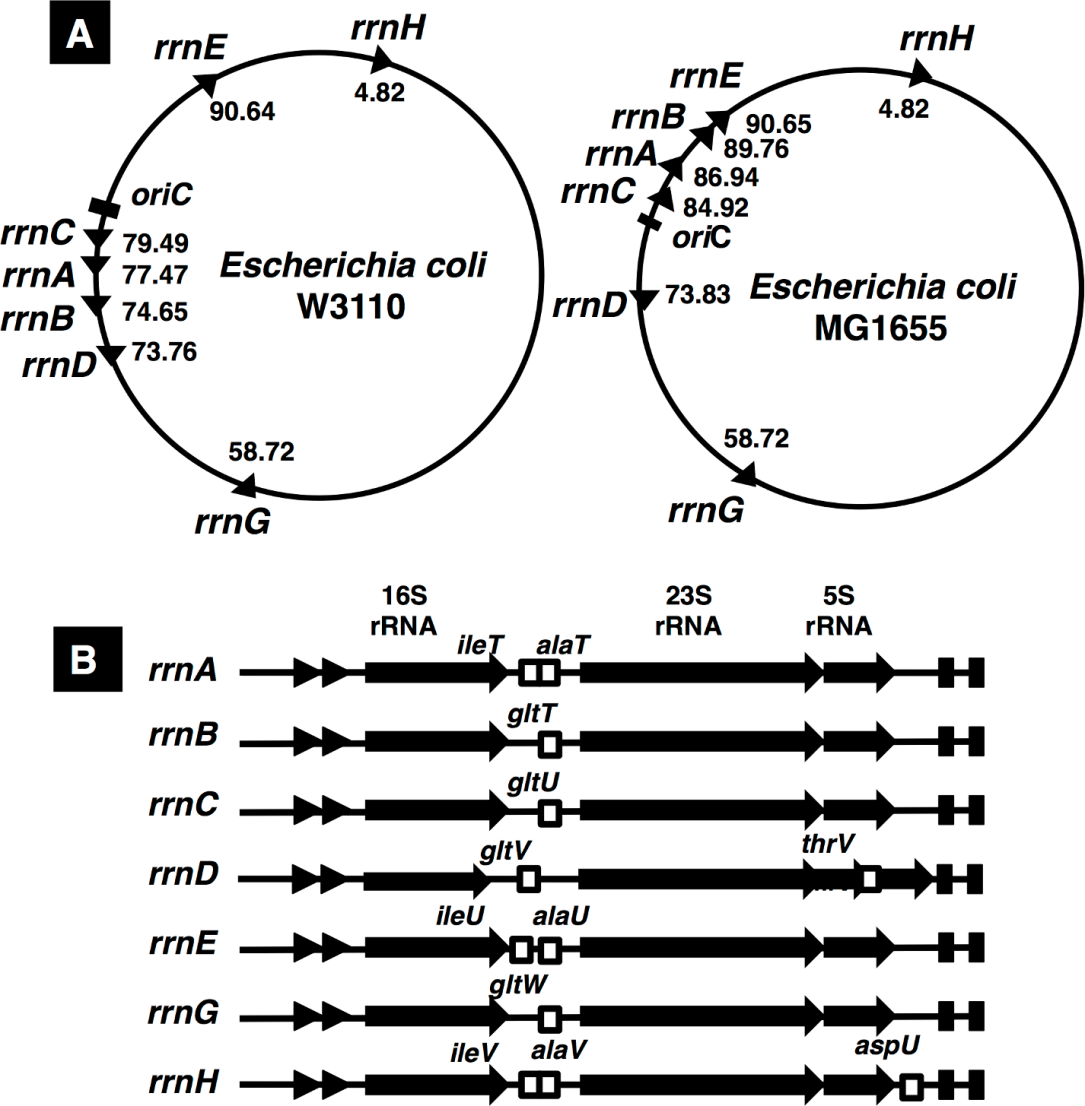
Gene organization of seven *rrn* operons in the *E*. *coli* K-12 genome. Seven rRNA operons exist in more than 2,000 *E*. *coli* strains so far sequenced [39]. Two K-12 strains, MG1655 [7] and W3110 [8], are widely used as the model *E*. *coli* strains. Strain W3110 carries an inversion between *rrnD* and *rrnE* operons [A], but the direction of transcription for all seven *rrn* operons are the same with that of DNA replication in both strains. The order of rRNA organization in seven *rrn* operons are the same, but each *rrn* operon contains one to three different tRNA genes [B]. Details are described in text.

Ribosomes are composed of 3 species of rRNA (16S, 23S and 5S rRNA) and a total of 22 species of ribosomal protein (r-proteins) for 30S ribosome and 35 species of r-proteins for 50S ribosomes. The syntheses of 3 rRNA species and all r-protein species are generally coordinated for efficient assembly of ribosomes. The genome of *Escherichia coli* K-12 W3110 contains seven rRNA operons, which are similar in the gene organization [7,8], each consisting of tandem promoters (P1 and P2), 16S rRNA gene, tRNA genes (tRNA^Ile^ and tRNA^Ala^ genes in the *rrnA*, *rrnE* and *rrnH* operons); and tRNA^Glt^ gene in the *rrnB*, *rrnC*, *rrnD* and *rrnG* genes); 23S rRNA genes; 5S rRNA gene; and at 3’ termini, tRNA^Thr^ gene in the *rrnD* and tRNA^Asp^ gene in the *rrnH* operon, respectively [9] (Fig 1). Each of the seven rRNA operons is transcribed from two promoters, upstream P1 and downstream P2, which are separated by approximately 120 bp in length [9]. P1 plays a major role in high-level synthesis of rRNA during exponential growth in nutrient-rich media while P2 accounts for basal-level synthesis of rRNA at low growth rate and in stationary phase of cell growth. Both *cis-*acting elements (AT-rich ‘UP element’ and GC-rich ‘discriminator’) within the promoter region and *trans*-acting protein factors (Fis, H-NS, Lrp and DksA) are involved in the regulation of rRNA synthesis [10,11,12]. In addition, two nucleotide factors, ‘alamone’ ppGpp (and pppGpp) [13–15] and ‘initiating nucleotide’ iNTPs [16], participate in this regulation [10,12,17].

Even though the sequences of seven *rrn* operons are almost identical, significant difference exists in the promoter sequence between seven tRNA operons, and the rRNA sequences encoded by seven *rrn* operons include operon-specific minor difference (see Discussion). Seven *rrn* operons in *E*. *coli* are located in noncontiguous sites around the genome and are transcribed in the same direction of genome replication [18]. The redundancy of rRNA operons in *E*. *coli* is considered to have evolved to support the high levels of ribosome production necessary for rapid growth rates [9,19,20]. All seven rRNA operons are needed for optimal adaptation to changing physiological conditions [21]. After systematic approaches of making *E*. *coli* strains with deletion of rRNA operons [22,23], the level of growth reduction was found to correlate with the deleted number of rRNA operons. The presence of even a single rRNA operon on the genome is able to produce as much as 56% of wild-type levels of rRNA [23]. The correlation between the number of *rrn* operons and the rate of cell growth was also confirmed by using a set of engineered rRNA opeon copy-number variants [24]. These findings indicated that *E*. *coli* harbors an excessive level of ribosomes, keeping a certain level of ribosome storage. In fact, unused ribosomes are stored in inactive forms by forming ribosome dimers after interaction with dimerization factors such as ribosome modulation factor RMF [25].

The seven rRNA copies have long been believed to be identical in the structure and function. This concept was challenged after analysis of the expression mode of seven rRNA operons in *E*. *coli*. Initial attempts were made to estimate the difference in the promoter activity of each rRNA operon using the protein reporter systems. Condon *et al*. [26] analyzed CAT (chloramphenicol acetyl transferase) expression by rRNA promoter-*cat* operon fusions while Hirvonen *et al*. [27] analyzed expression of rRNA promoter-*lacZ* operon fusion. Since RNA genes lack the signal for translation, the reporter gene-associated SD sequence was used for expression of the reporter proteins in these studies. Nevertheless, these pioneering studies suggested the growth rate- and growth phase-dependent difference in the expression pattern between seven rRNA operons.

Here we demonstrate the intrinsic characteristics for seven rRNA promoters by using the two fluorescent protein (GFP/RFP) reporter system, that was developed for accurate measurement the promoter strength and regulation of protein-coding genes [28]. Approximately 500 bp-long segment upstream from 5’ end of 16S rRNA was inserted, after addition of SD sequence for reporter translation, into this pBRP promoter assay vector yielding the improved vector pBRS to be used for the promoter assay of RNA genes. These direct fusions between each rRNA promoter-GFP coding sequence, a kind of translational fusion, allowed more accurate estimation of the strength and regulation of rRNA promotors. Results herein described indicate that: the promoter activity is maximum for the *rrnE* operon; but the order of promoter strength between other six *rrn* operon promoters is different depending on the growth conditions. The molecular basis of the difference in promoter strength and regulation is discussed in relation of possible difference in the physiological role of each *rrn* operon.

## Results

### Construction of the promoter assay system for all seven rRNA operons

*E*. *coli* contains a total of seven rRNA operons, of which five (*rrnC*, *rrnA*, *rrnB*, *rrnE* and *rrnH* on this order from the replication origin) are in clockwise direction and two (*rrnD* and *rrnG*) in anti-clockwise direction (Fig 1). All seven rRNA operons contain tRNA genes: both Ile-tRNA and Ala-tRNA genes within spacer between 16S and 23S rRNA genes for *rrnA*, *rrnE* and *rrnH* operons; one Glu-tRNA gene between 16S and 23S rRNA genes for *rrnB*, *rrnC*, *rrnD* and *rrnG* rRNA operons. In addition, downstream of 23S rRNA gene, one Thr-tRNA gene in the *rrnD* operon and one Ala-tRNA gene in the *rrnH* operon. The Glu tRNA gene is not present outside the *rrn* operons, implying that one evolutionary force for maintenance of the *rrnB*, *rrnC*, *rrnD* and *rrnG* operons is the inclusion of Glu-tRNA gene within these composite *rrn* operons.

**Fig 2:**
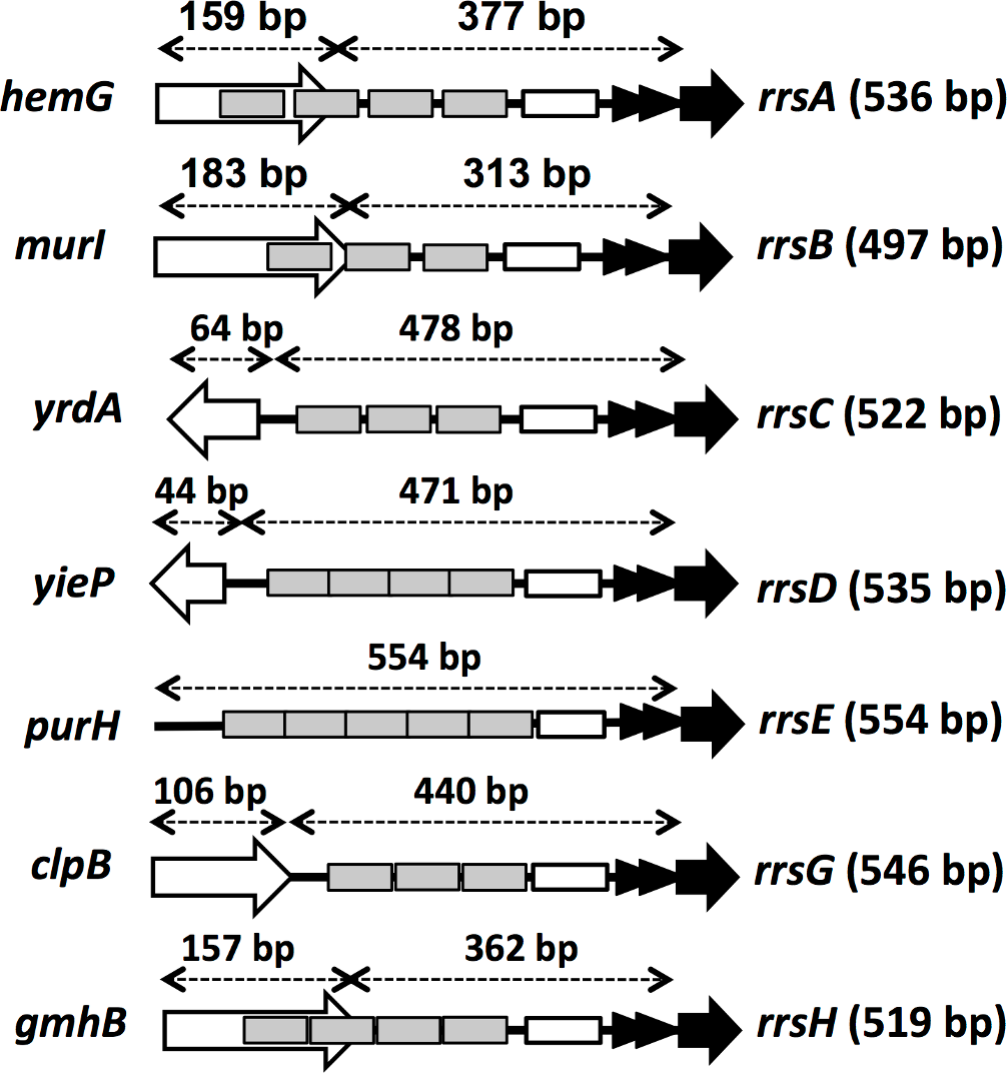
Promoter-region sequences of seven *rrn* operons used for construction of the promoter assay vectors. For construction of the promoter assay vectors of *rrn* operons, a total of approximately 500 bp-long sequence upstream from 5’ terminus of 16S rRNA gene, as indicated above each DNA lane, were PCR-amplified using specific set of primers (for primer sequences see S1 Table) and inserted into pGRS vector, a modified form of pGRP [28], containing an SD sequence at the junction of GRF-coding sequence. Open box and closed boxes on each probe represent the relative location of UP element and predicted Fis-binding sites, respectively (see Fig 8). Triangles downstream of the UP element indicate two promoters, upstream P1 and downstream P2. The number of Fis sites on the *rrnE* promoter was suggested to be more than those hitherto identified (see Fig 3). The whole length used for the construction of pGRS vector is described in parenthesis at right-side end of each lane.

**Fig 3:**
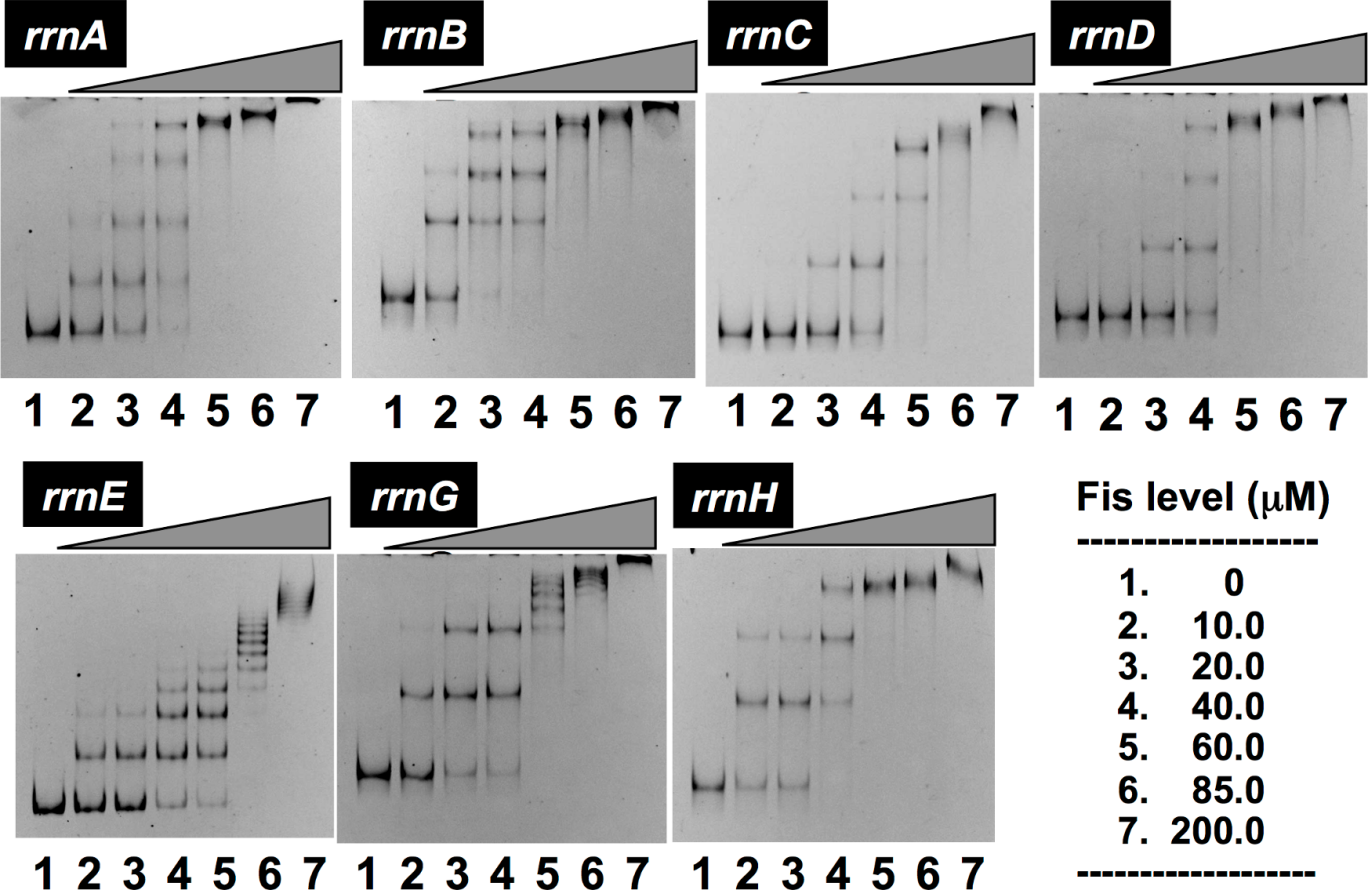
Gel shift assay of Fis binding to promoter DNA probes of seven *rrn* operons. Gel shift assay of Fis-binding sites was performed under the standard procedure as described in Materials and Methods using each of seven *rrn* promoter probes as shown in Fig 2 and increasing amounts of purified Fis protein. With the *rrnE* and *rrnG* promoters, Fis exhibited high-level of cooperative binding, forming multiple ladders.

On each rRNA promoter, three to five Fis-binding sites have been proposed (Fig 2). To confirm these proposals, we analyzed the number of Fis-binding sites along each 500 bp-long rRNA promoter segment. Based on the gel shift assay, the number of Fis-binding sites on *rrnA*, *rrnb*, *rrnC*, *rrnD* and *rrnH* were estimated to be the same as reported [27], but that on *rrnE* increased from 5 to more than 8, and that on *rrnG* increased from 3 to more than 6 (Fig 3). Based on the gel shift pattern, the apparent increase in Fis-binding sites on these two promoters was suggested to be attributable to the cooperative binding of Fis at high protein concentrations.

**Fig 4:**
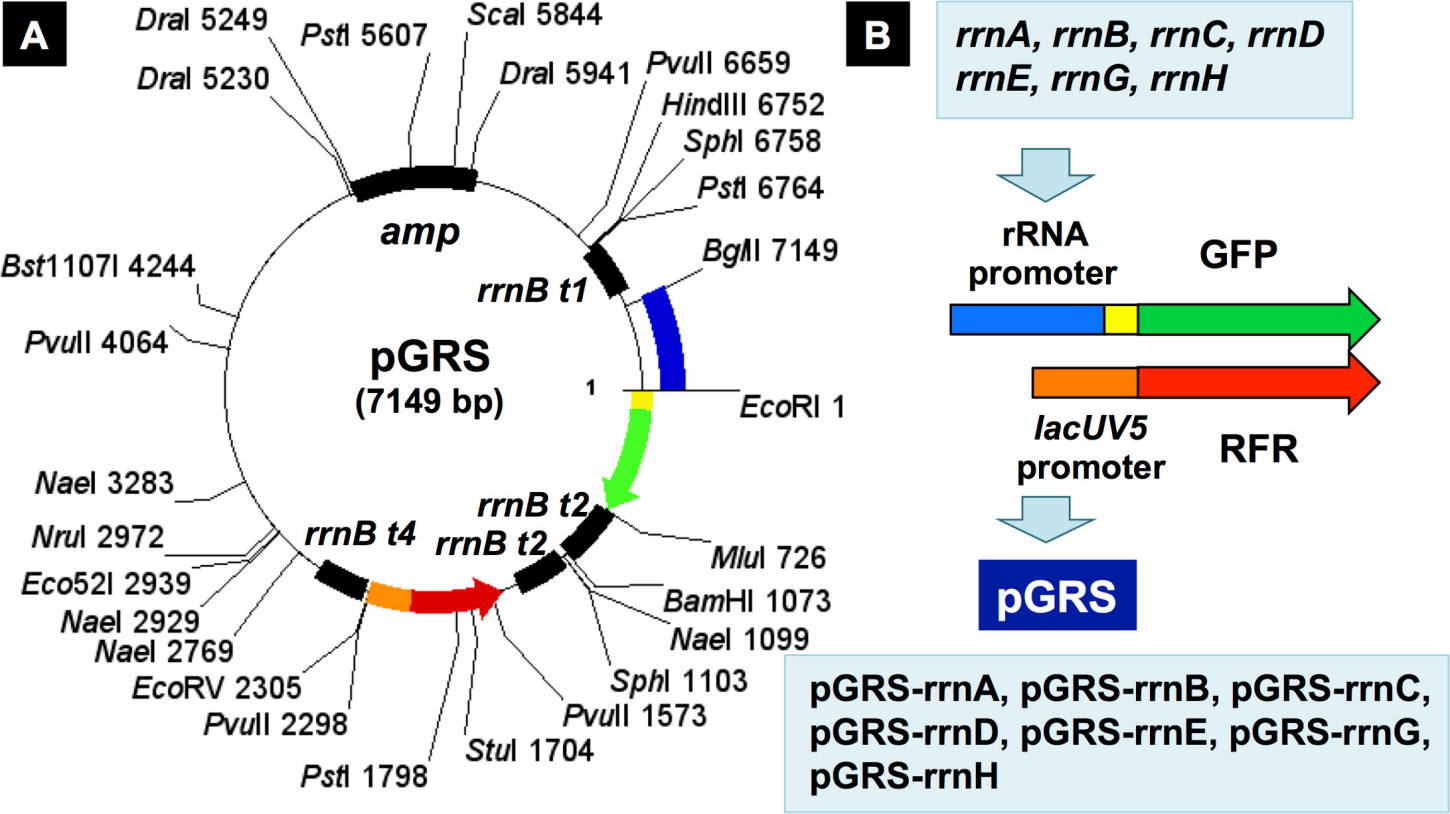
Map of the two-fluorescent reporter vector for the promoter assay of seven *rrn* operons. For determination of the promoter strength and regulation of seven *rrn* operons, approximately 500 bp-long DNA fragments (indicated by blue; for details see Fig 2) covering each of the *rrn* promoters, as shown in Fig 2, were PCR-amplified and inserted into pGRS vector (the modified version of pGRP vector with an inserted SD sequence (indicated by yellow).

Previously we constructed the quantitative promoter assay systems using GFP-RFP two-fluorescent protein reporters [28]. For measurement of RNA promoter strength without SD sequence for translation, we modified the original pGRP vector by adding a SD sequence prior to the coding sequence of GFP (Fig 4). This newly constructed pGRS vector (the modified pGRP with SD sequence added) was used in this study for measurement of the strength and regulation of rRNA promoters. Approximately 500 bp-long sequence upstream the initiation site of each *rrn* promoter (see Fig 2) was cloned into pGRS vector between *Bgl*II and *EcoR*I sites so as to insert them prior to the SD sequence. The resulting pGRS series vectors (pGRS-rrnA to pGRS-rrnH) (Table 1) allowed the detection of strength of each *rrn* promoter by measuring the relative level of GFP and RFP expression levels (Fig 4).

**Table 1:** Bacteria and plasmids used in this study.

Bacterial strain	Genotype
*Escherichia coli* W3110	*rph*-1 INV (*rrnD*, *rrnE*)
*Escherichia coli* DH5α	*supE*44 *lacU*169 *deoR nupG* Δ*(lacZYA-argF*) *hsdR17 recA1endA1 gyrA96 thi-1 relA*
*Escherichia coli* BL21(DE3)	*dcm ompT hsdS gal* (λDE3)
*Escherichia coli* BW25113	*rrnB ΔlacZ4787 hsdR514 ΔaraBAD567 rph-1*
*Escherichia coli* JW1225	BW25113 *hns*
*Escherichia coli* JW3229	BW25113 *fis*
Plasmid	Construction
pBR322	
pGRP	Promoter assay vector (Shimada T et al. [28])
pGRS	SD within pGRP
pGRS-rrnA	*rrnA* promoter within pGRS
pGRS-rrnB	*rrnB* promoter within pGRS
pGRS-rrnC	*rrnC* promoter within pGRS
pGRS-rrnD	*rrnD* promoter within pGRS
pGRS-rrnE	*rrnE* promoter within pGRS
pGRS-rrnG	*rrnG* promoter within pGRS
pGRS-rrnH	*rrnH* promoter within pGRS
pGRS-lacUV5	*lacUV5* promoter within pGRS

### Growth phase-coupled changes in the promoter activity of seven rRNA operons

**Fig 5:**
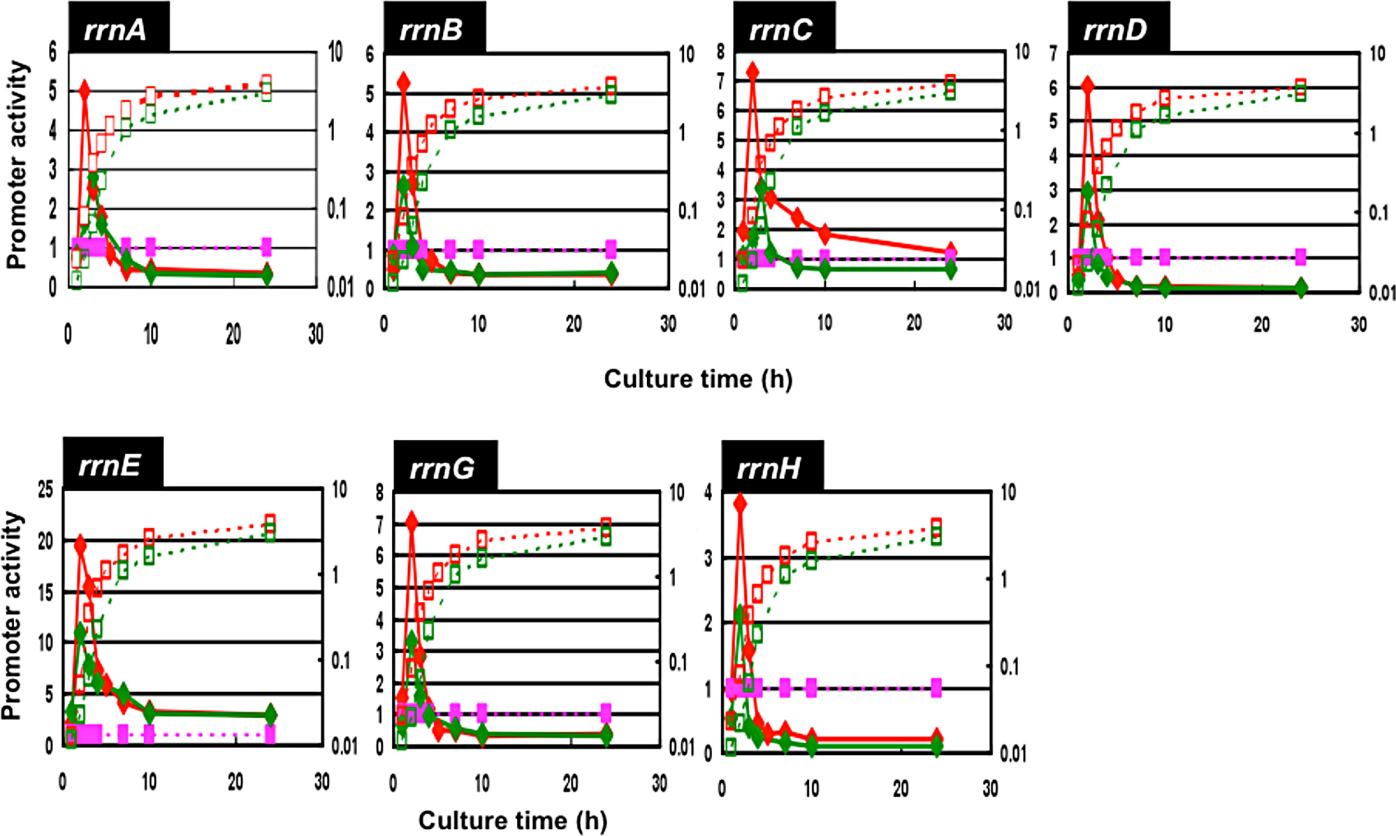
Growth phase-dependent variation of the promoter activity of seven *rrn* operons: LB and M9-Glc-CA growth. Promoter activity of seven *rrn* operons was determined using a series of pGRS vector, two fluorescent reporter assay vectors (for the plasmid map see Fig 4), which were transformed into *E*. *coli* W3110 type-A. Transformants were grown at 37°C in LB (red) and M9+glucose+casamino acids (green). The growth curve (dotted lines) was determined by measuring turbidity the promoter activity (straight lines) was determined by measuring the fluorescent intensity and is shown as the relative values with the reference of *lacUV5*-directed activity. Purple symbols and lines represent the *lacUV5* promoter-directed GFP level that was set at 1.0.

Using the newly developed vectors for the assay of RNA promoters thus constructed, we first determined growth phase-dependent changes in the promoter activity of each rRNA operon. Each of seven pGRS vectors was transformed into wild-type *E*. *coli* K-12 W3110 A-type with the intact *rpoS* gene [29]. A reference vector pGRS-*lacUV5* with *lacUV5* promoter inserted prior to both GFP and RFP coding sequences was also transformed into *E*. *coli* W3110. Transformants were grown in LB and the levels of GFP and RFP fluorescence were measured at every hour up to 30 hr (Fig 5A). The expression level of RFP under the control of *lacUV5* stayed almost constant throughout growth phases as measured using GFP/RFP ratio in good agreement with the systematic measurement of promoter activity using the original pGRP vector [28]. To cancel possible fluctuation of the plasmid copy numbers, the level of rRNA promoter-directed expression of GFP was calculated by measuring the ratio of GFP (under the direction of test rRNA promoter) and RFP (under the direction of reference *lacUV5* promoter). The level of promoter activity was the highest in the exponential growth phase at 2 hr after inoculation of the preculture to fresh media for all seven rRNA promoters and then decreased upon entry into stationary phase. The high-level activity of *rrn* promoters agrees with the high-level production of ribosomes in growing phase. The rate of decrease in promoter activity was relatively slow for pGRS-rrnC (*rrnC* promoter) and pGRS-rrnE (*rrnE* promoter), and thus the GFP fluorescence still remained at detectable levels even at 20 and 30 hr after inoculation of the over-night preculture to a fresh medium.

### Growth rate-coupled changes in the promoter activity of seven rRNA operons

**Fig 6:**
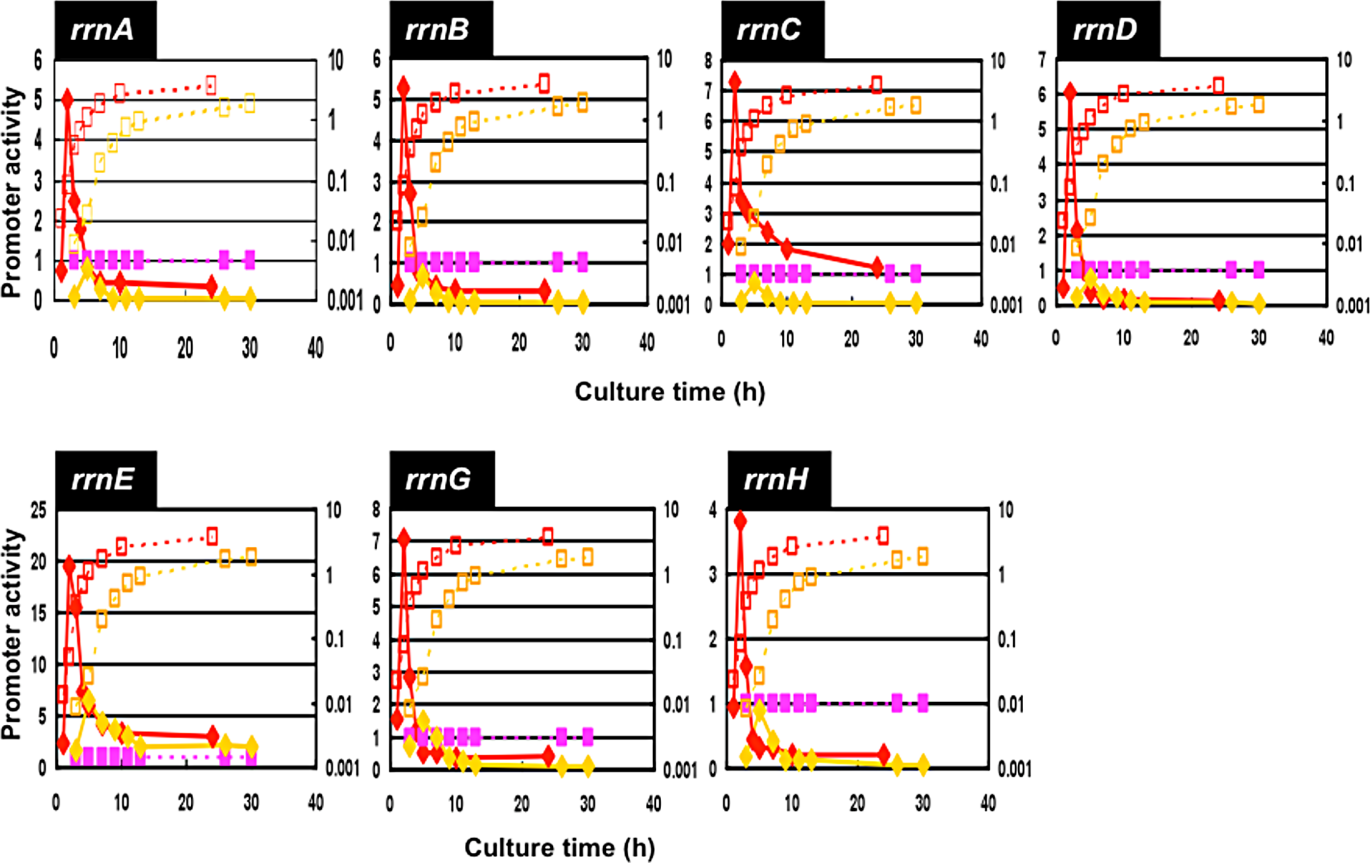
Growth phase-dependent variation of the promoter activity of seven *rrn* operons: LB and M9-Glc growth. Promoter activity of seven *rrn* operons was determined for the culture grown in LB (red) and M9-glucose (orange) media. Growth curve and promoter activity were measured as in Fig 5, and are shown in red (LB) and orange (M9-Glc).

**Fig 7:**
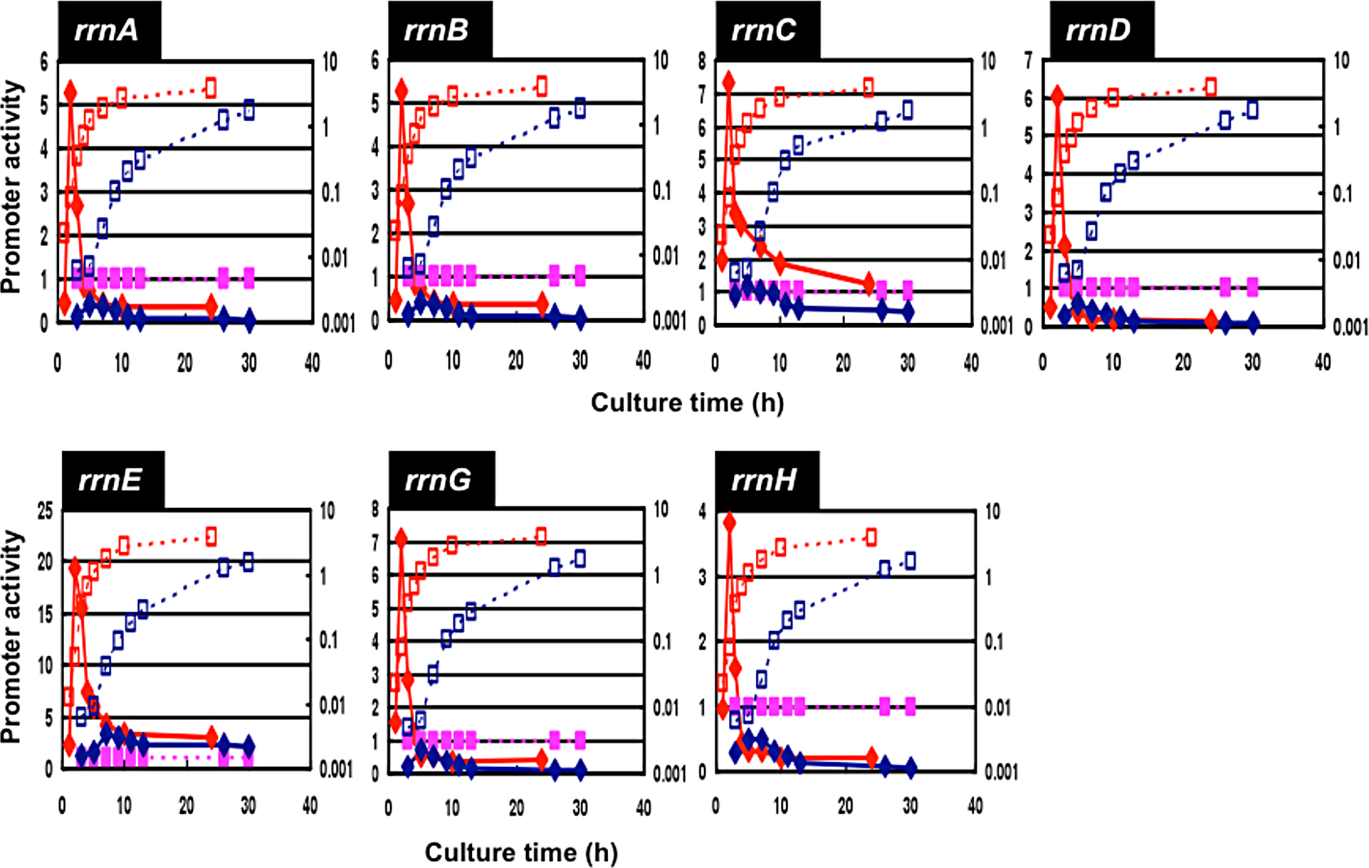
Growth phase-dependent variation of the promoter activity of seven *rrn* operons: LB and M9-Gly growth. Promoter activity of seven *rrn* operons was determined for the culture grown in LB (red) and M9-glycerol (blue) media. Growth curve and promoter activity were measured as in Fig 5, and are shown in red (LB) and blue (M9-Gly).

The level of *rrn* promoter activity was then compared for cells growing at different rates. For this purpose, transformants carrying each pGRS vector were grown in four different media: a) LB (Luria-broth); b) M9-0.4% glucose plus casamino acid; c) M9-0.4% glucose; and d) M9-0.4% glycerol. The growth rates were: 1.8–1.9 doublings per hr (a); 1.3–1.35 doublings per hr (b); 0.6–0.65 doublings per hr (c); and 0.4–0.45 doublings per hr (d), respectively. For each culture, the promoter activity was measured as the relative value to that of reference *lacUV5* promoter-directed activity (purple line for each panel in Figs 5–7) at various times up to 30 hr after inoculation of the preculture to each fresh medium.

In the growth phase-dependent change in the promoter activity for the M9-glucose-casamino acid culture is slightly different from that in LB culture (Fig 5, red line). As in the case of LB culture, the maximum activity was observed at the 2 hr for all seven rRNA promoters (Fig 5, green line). The growth phase-coupled decrease in the activity of *rrn* promoters was observed for the slowly growing culture in M9-0.4% glucose (Fig 6) and M9-0.4% glycerol (Fig 7). In both of these slowly growing cultures, the maximum promoter activity was observed at the culture time 5 hr. The delay in the appearance of *rrn* promoter activity is mainly attributable to the decreased rate of cell growth in nutrient-poor media.

**Fig 8:**
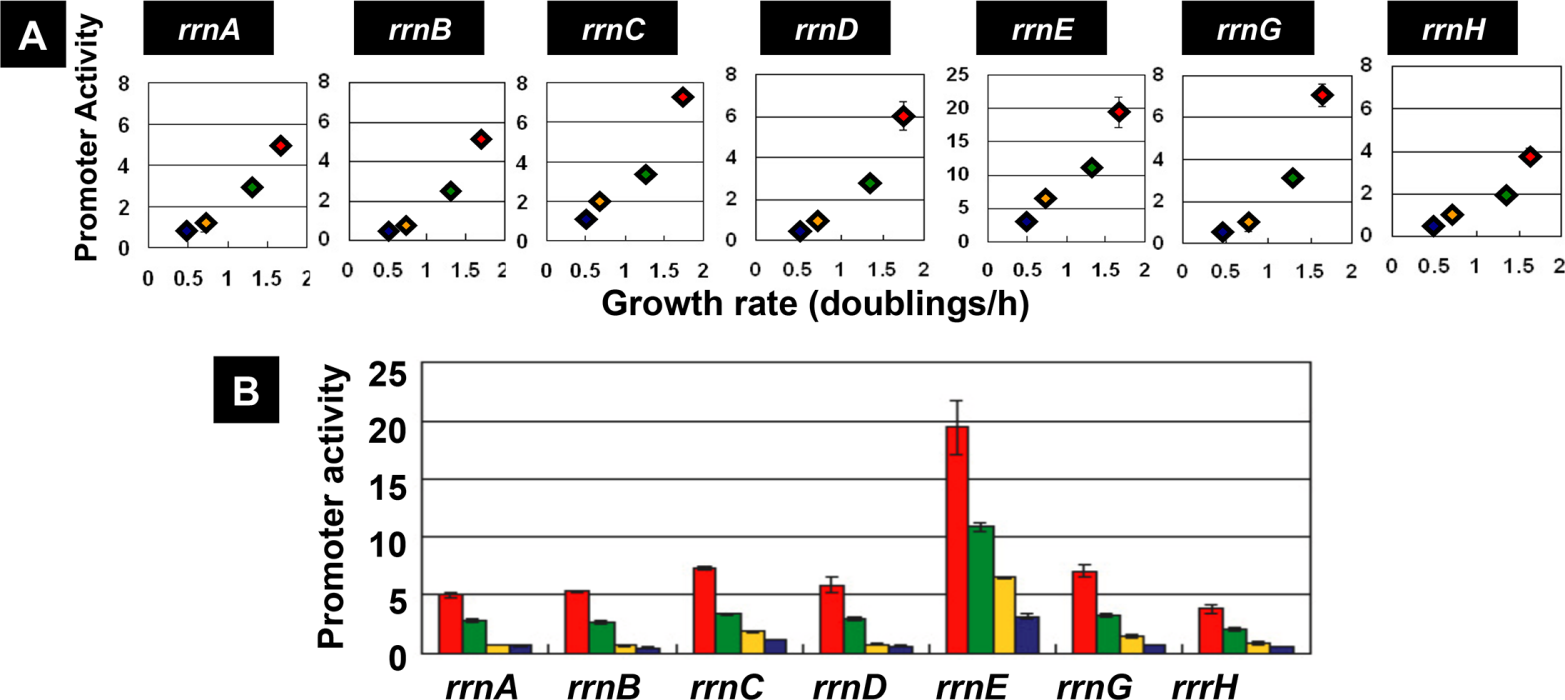
Growth rate-dependent variation of the promoter activity of seven *rrn* operons. The promoter activity of seven *rrn* operons of *E*. *coli* was separately determined as described in Fig 5. [A] The ratio between the cell growth rate in four different culture media and the highest level of promoter activity in the middle of exponential growth phase is plotted for each of seven *rrn* promoters. [B] The highest level of promoter activity in four different cultures is shown for each of seven *rrn* operon promoters. The activity of *rrnE* promoter was the highest under all the culture conditions employed.

The promoter activity was calculated at different times for all four cultures grown in different media. The highest levels of promoter activity are compared between these different cultures (Fig 8A). Between seven rRNA promoters, the activity at the peak was the highest for *rrnE*, and the lowest for *rrnH*, the level being *rrnE* > *rrnG* > *rrnC* > *rrnD* > *rrnB* > *rrnA* > *rrnH* in decreasing order. The difference in promoter strength ranged 6–7 fold between the strongest *rrnE* and the weakest *rrnH*. Nevertheless the order of promoter activity in different media was the same between seven *rrn* promoters, showing the decreasing order: LB (a) > M9-glucose-casamino acid (b) > M9-glucose (c) > M9-glycerol (d) (Fig 8B).

### The promoter activity of all seven rRNA operons in mutants lacking Fis or H-NS

Up to the present time, several regulatory factors have been indicated to be involved in regulation of seven *rrn* promoters, including DNA UP element, transcription factors Fis, Lrp and H-NS, and nucleotide effectors ppGpp and iNTP (initiating nucleotides ATP or GTP). Nucleoid of *E*. *coli* contains about 10 species of the DNA-binding protein with both architectural and regulatory roles [29,30], of which two proteins, Fis and H-NS, have been proposed to play major roles in expression of *rrn* operons [10,16]. The influence of Fis and H-NS on individual *rrn* operons is not yet fully understood. As an attempt for elucidation of the regulatory factors that influence the growth phase-dependent pattern of each promoter activity, we analyzed in this study the activity of each *rrn* promoter in mutants *E*. *coli* lacking the proposed potent regulators Fis and H-NS.

**Fig 9:**
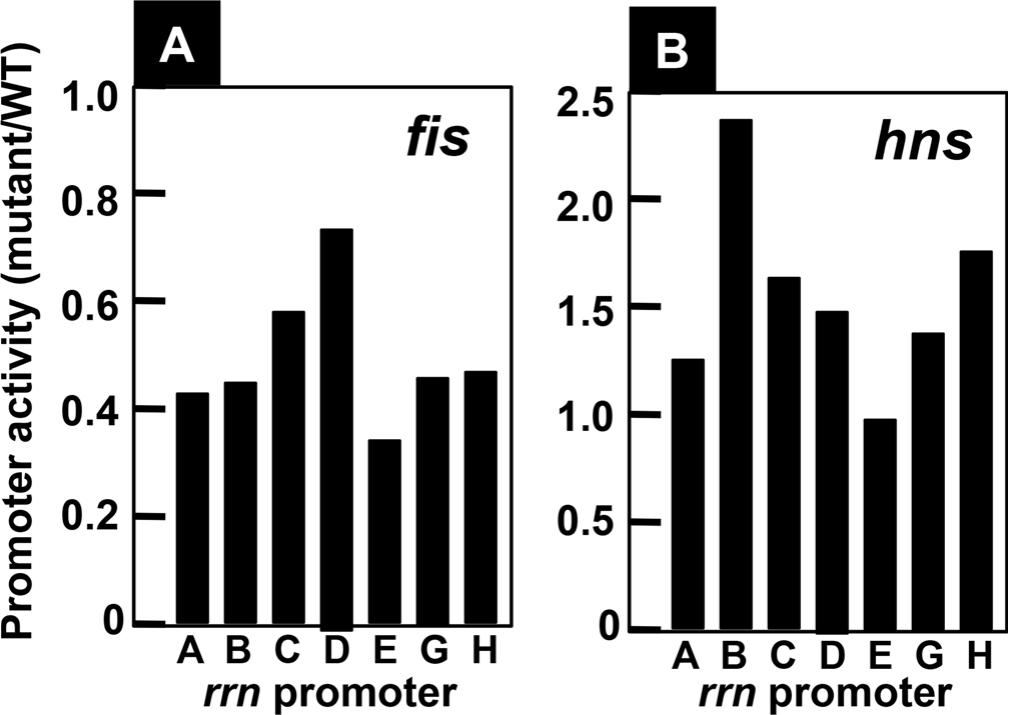
Promoter activity of seven *rrn* operons in mutants lacking the *fis* or *hns* genes. Promoter activity of seven *rrn* operons was determined using the double-fluorescent protein reporter system as in Fig 5. [A] The promoter assay of seven *rrn* operons was performed using wild-type and a mutant lacking the *fis* gene. The ratio of maximum promoter activity between the *fis* mutant and wild-type is shown for each of seven *rrn* promoters. The maximum reduction in the absence of Fis was observed for the *rrnE* promoter. [B] The promoter assay of seven *rrn* operons was carried out for wild-type and a mutant lacking the *hns* gene. The ratio of maximum promoter activity between the *hns* mutant and wild-type is shown for each of seven *rrn* promoters. The maximum activation in the absence of H-NS was observed for the *rrnB* promoter that carries a strong binding site for H-NS [37]. The assay was repeated at least twice and the fluctuation level was less than 10%.

Fis plays a major role in high-level expression of the growth-coupled genes including the *rrn* operons [31]. In the absence of Fis, the growth rate decreased significantly due to the reduction of rRNA production (data not shown). The influence of Fis deletion was compared between seven rRNA promoters (Fig 9A). The level of Fis deletion reduced, to various extent, the expression of *rrn* operons as measured by the reporter assay, in good agreement with the reduction of cell growth. The reduction in promoter activity was the maximum for *rrnE* (less than 35% the level of wild-type value in the presence of Fis) and the minimum for *rrnD* (about 75% the level of wild-type value). The number of Fis-binding sites is the highest for *rrnE* (see Fig 3), indicating the influence of Fis deletion correlates with the number of Fis-binding sites. This finding supports the prediction that the high-level of promoter activity for *rrnE* is, at least in part, to the high-level binding of activator Fis.

Nucleoid protein H-NS is known as the general silencer of a large number of unused genes and horizontally acquired genes [30,32]. By using the genomic SELEX screening system [32], we have identified a total of 987 H-NS binding sites on the *E*. *coli* genome [33]. Between seven *rrn* operon promoter regions, a high-level of H-NS binding was identified on three *rrn* promoters, *rrnA*, *rrnB* and *rrnH* [34]. In good agreement with the presence of H-NS binding sites, the increase in promoter activity in the *hns* mutant was the highest for the *rrnB* promoter, followed by the *rrnH*, *rrnC* and *rrnD* promoters (Fig 9B). Noteworthy is that virtually no influence of H-NS deletion was observed for the *rrnE* promoter, which was maximally activated by Fis in exponentially growth phase (Fig 9A). Since H-NS interferes, as the general silencer, with the binding of RNAP and Fis to these promoters, the low activity of *rrnA*, *rrnB* and *rrnH*, in particular in the stationary phase, might be attributable to the silencing effect by H-NS. These observations altogether confirmed the notion that the expression of seven *rrn* operons is indeed under different control by two global regulators Fis and H-NS.

## Discussion

### Multiplicity of the *rrn* gene copies

rRNA genes have been widely used for the taxonomic assignment of individual organisms and the estimation of evolutionary history of species. The level of sequence heterogeneity among *rrn* operons influences the accuracy of species identification [20,35]. The existence of multiple rRNA operons within a single bacterial species is widely identified, ranging from one to 15 copies per genome [20]. The presence of seven rRNA operons in *E*. *coli* is preserved among a total of more than 2,000 strains so far sequenced [36]. The redundancy of seven rRNA operons in *E*. *coli* must have evolved to support the high-level production of ribosomes and thereby the high-level of cell growth [9,19]. The multiplicity of *rrn* operons has been considered to be primarily a mechanism for maintaining the appropriate amount of rRNA in a cell. One genetic system to keep the multiple copies of rRNA gene is the inclusion of specific tRNA genes within all seven rRNA operons. In the spacer region of 16S and 23S rRNA genes, one Glu-tRNA gene exists in each of the *rrnB*, *rrnC*, *rrnD* and *rrnG* operons and both Ala-tRNA and Ile-tRNA genes exist in the *rrnA*, *rrnE* and *rrnH* operons (see Fig 1). In addition, in the 3’-proximal region after 23S rRNA gene, the *rrnD* operon includes one Thr-tRNA gene between two 5S rRNA genes, and the *rrnH* operon includes one Asp-tRNA gene after 5S rRNA gene. Among these four species of tRNA, the genes encoding Glu-tRNA are present only the *rrnB*, *rrnC*, *rrnD* and *rrnG* operons, indicating at least one of these *rrn* operons is essential for cell growth.

The high-level conservation of rRNA-coding sequence indicates the participation of rRNA in important functions associated with ribosomes. The function of ribosomes as the apparatus of translation is unique: unlike other cellular enzymes, the ribosomes are ribozymes [37–39], in which rRNAs play key roles in protein synthesis. All three species of rRNA in ribosomes contribute, to various extents, in the rRNA functions, including peptide bond formation, RNA-RNA interaction with mRNA and tRNAs, intra- and inter-molecular interaction of rRNAs, RNA-protein interaction with a number of r-proteins and translation factors, and interaction with ribosome-targeted antibiotics. The presence of multiple rRNA gene copies is similar to those of the alloenzymes and alloproteins. At present, several alloproteins have been identified in *E*. *coli*, which are variant forms of a single and the protein, each being encoded by an independent allele. In the pathway of gene expression, for instance, two molecular species of elongation factor Tu (EF-Tu) exist, which are encoded by two different genes, *tufA* and *tufB*, together contributing to the highest level expression, 5–10% of total proteins or 10-times more than ribosomes, in *E*. *coli* [40]. Two EF-Tu proteins differ only at a single position at the C-terminus, Gly394 for TufA and Ser394 for TufB [41]. In the metabolic pathways, a number of allozymes (also called isozymes) have been identified. In some cases, however, the gene products are post-translationally modified. For instance, three forms of alkaline phosphate differ differing in the presence and absence of N-terminal Arg [42,43].

In addition to the inclusion of different tRNA gene(s) in each of the composite *rrn* operons, rRNAs encoded by seven *rrn* operons in *E*. *coli* must play specific and different roles in rRNA functions and/or in maintenance of all seven *rrn* operons in evolutionary time scale.

### Possible functional heterogeneity of rRNA encoded by seven *rrn* operons

**Fig 10:**
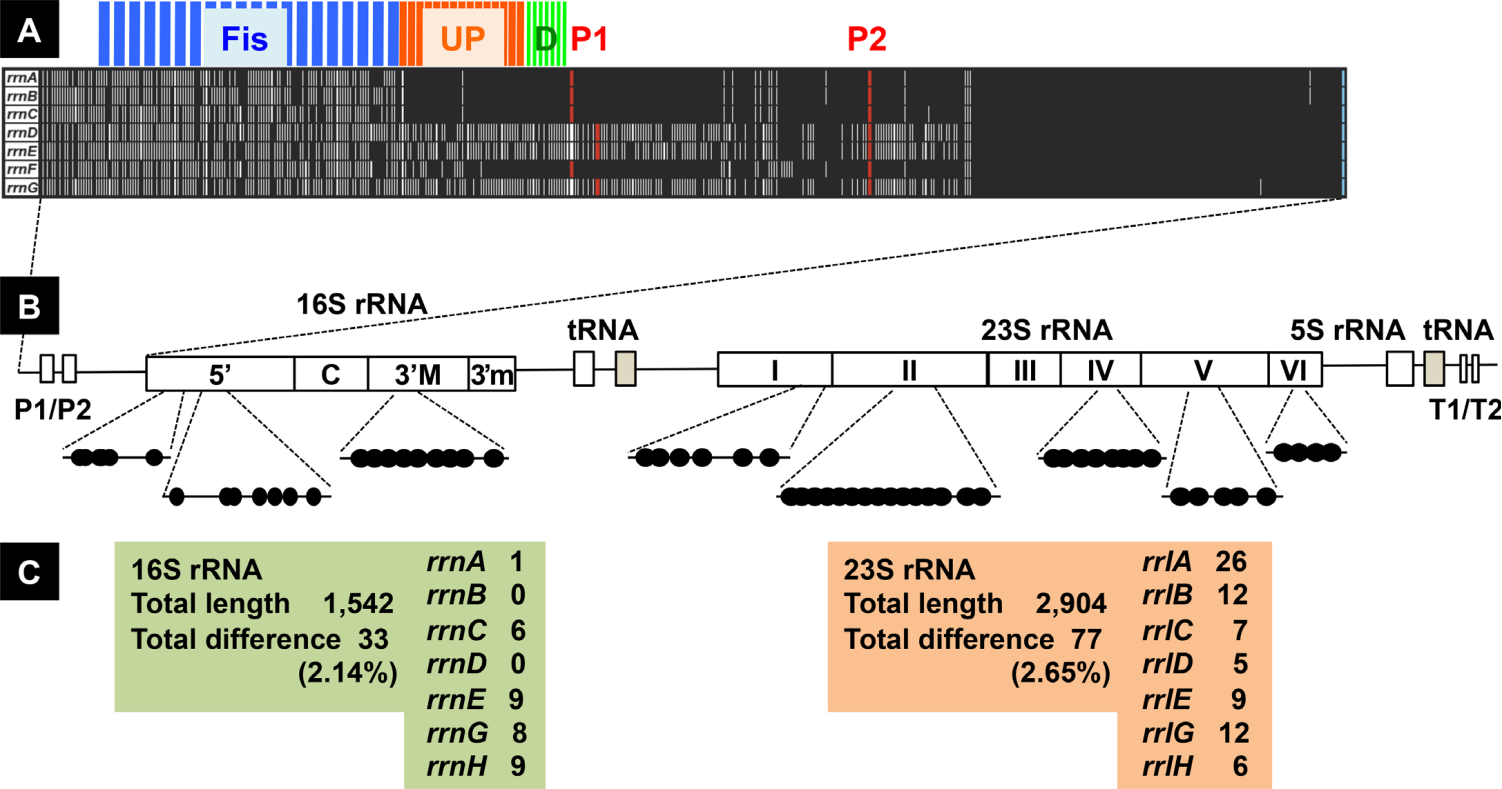
Sequence difference between seven *rrn* operons. [A] Upper panel shows the 500 bp-long sequences upstream of the start site of mature 16S rRNA from all seven *rrn* operons. The sequences conserved between seven *rrn* operons are shown in black but the positions that differ from the conserved sequences are shown in white. Red bars indicated transcription start sites of upstream P1 and downstream P2 promoters. Marked variation was identified in the promoter regions, in particular, upstream from P1 promoter. [B] The positions of sequence variation within 16S and 23S rRNA are shown along the gene organization of *rrn* operons. A total of 33 and 77 differences were identified in 16S rRNA and 23S rRNA, respectively. [C] The number of bases that are different from the conserved sequence are shown for 16S and 23S rRNA for each *rrn* operon.

After genome sequencing, a considerable level of variation was found to exist between 16S, 23S and 5S rRNAs encoded by seven rRNA operons in *E*. *coli*. Even though the diversity between each rRNA species within a single and the same species is limited, the combined numbers of sequence difference between seven *rrn* operons are 2.14% for 16S rRNA, and 2.65% for 23S rRNA (Fig 10; for details see S1 Fig). The sequence diversity of 16S rRNA is the maximum for *rrsH* (0.58%) while that of 23S rRNA is the maximum for *rrlA* (0.89%). Further expansion of the sequence diversity must have been prevented through the homogenization process by homologous recombination. Based on the location of sequence diversity between seven *rrn* operons, we can predict the homogenization of 16S rRNA through recombination between *rrsC*, *rrsE* and *rrsG* (S1A Fig). Likewise, the homogenizations of 23S rRNA must have taken place at least two regions: 5’-proxial region between *rrlB*, *rrlE* and *rrlG* and 3’-proximal region between *rrlB*, *rrlD* and *rrlG* (S1B Fig).

Since rRNAs carry the important catalytic activities, the presence of sequence difference, albeit at low levels, within the rRNA genes between seven rRNA operons implies possible difference in the functions and/or specificity of rRNA molecules. The sequence difference summarized in Fig 10 is, however, limited within the regions that are not involved in the catalytic functions of peptide bond formation [44,45]. The sequence difference is also not observed at the target nucleotides of post-transcriptional modification such as base methylation and at the sites involved in binding of ribosome-targeted antibiotics [49]. On the contrary, the sequences of high-level variation appear to be exposed on the surface, playing roles in contact with r-proteins and other ribosome-associated protein factors [46]. For instance, the nucleotide 79–93 region of 16S rRNA (see S1A Fig), differing between two groups (one group consisting of *rrsA*, *rrsB*, *rrsD* and *rrsH*, and another including *rrsC*, *rrsE* and *rrsG*), is located within 5’ domain of 16S rRNA for direct contact with r-proteins S17, S20 and S4 [47]. This region forms one of the bridges between 30S and 50S ribosomes [39]. Likewise the nucleotide 1869–1884 region of 23S rRNA is altered only the *rrlA* gene (see S1B Fig). This region is located within domain II of 23S rRNA, forming another contact bridge between 30S and 50S subparticles [39]. Even though the essential sequences for the catalytic function of rRNA are conserved between seven *rrn* operons, the difference in the sequence for 30S-50S bridging implies alteration in the affinity and specificity of ribosome assembly between 16S and 23S rRNAs encoded by seven *rrn* operons. Difference in the assembly mode between rRNA species must lead to altered expression of *rrn* operon through feedback regulation [48]. Along this line, it is worthwhile to check possible difference in the function of rRNA molecules encoded by each of seven rRNA operons.

### Differential expression of seven rRNA operons

In contrast to the low-level variation in rRNA sequences between seven rRNA operons, higher levels of the sequence variation exist within both the promoter (Fig 10) [9,49] and the spacer regions [50]. Previous studies using reporter assays of transcriptional fusion indicated different regulation between seven *rrn* operons [21,27]. Here we confirmed differential regulation of seven *rrn* operons using a newly developed translational fusion between each *rrn* promoter and the fluorescent protein-coding sequence (see Figs 4–9). Transcription of rRNA is the rate-limiting step in ribosome biosynthesis in *E*. *coli*. When the demand for protein synthesis is high during rapid growth, the rRNA promoters account for more than 60% of total cellular transcription [4]. The majority of transcription originates from P1 promoter in growing cells. Each P1 promoter contains an AT-rich sequence called the upstream (UP) element located upstream of the promoter -35 element [51,52]. UP elements interact with the C-terminal domain of the alpha subunit of RNA polymerase, leading to increase in rRNA transcription. Alpha subunit CTD binds to the minor groove of the UP element DNA, contacting both bases and phosphodiester backbone [53,54]. Upstream of the P1 promoter in all seven *rrn* operons, there is an activator region including multiple sites of Fis binding [55,56], which are located upstream of the UP element for the P1 promoters (Fig 10). Fis binding to these sites increases transcription of rRNA operons [27,51]. The Fis protein stimulates *rrn* P1 promoters by making protein-protein contacts with the alpha CTD [57,58]. Fis concentrations are very low at low growth rates and in stationary phase [59,60]. Fis and UP element together account for the high activity of each of the *rrn* P1 promoters, although Fis and UP make slightly different contributions, depending on the promoter [27]. The P2 promoter is considered to be constitutive, working in expression of low-level of rRNA in slowly growing cells in poor media and in stationary phase [10,61]. Since the binding of more than five Fis molecules was indicated to the *rrnE* promoter (see Fig 3), the high-level activity of these promoters could be due to co-operative binding of multiple Fis molecules. On the other hand, we have identified, by using genomic SELEX screening system, H-NS binding only on to three *rrn* promoters, *rrnA*, *rrnB* and *rrnH* [34]. Since H-NS interferes, as the general silencer, with the binding of RNAP and Fis to these promoters, the low activity of *rrnA*, *rrnB* and *rrnH*, in particular in the stationary phase, might be attributable to the silencing effect by H-NS. The intracellular level of Fis is the maximum in exponentially growing phase and decreased to virtually undetectable level in the stationary phase [59,60] while H-NS remains as a major population of nucleoid proteins in the stationary phase. Since the relative level of StpA, an analog of H-NS, increases over the H-NS level in the stationary phase [30], it is worthwhile to examine possible silencing role of StpA on *rrn* expression in the stationary phase.

## Materials and Methods

### Bacterial strains and culture conditions

*Escherichia coli* K-12 W3110 type-A with the intact *rpoS* gene [62] was used throughout this study (Table 1). The culture media used were: LB (Luria-Broth), and M9 minimal supplemented with 0.4% glucose-casamino acid, 0.4% glucose or 0.4% glycerol.

### Construction of promoter assay vectors

For quantitative measurement of the promoter activity *in vivo*, two types of fluorescent protein genes, one for the red fluorescent protein dsRed (Clontech) (referred to as RFP in this paper) and the other for the green fluorescent protein eGFP (Clontech) (referred to as GFP in this paper), were inserted into a single vector. The RFP gene was under control of reference promoter *lacUV5*, and the GFP gene was under control of a test promoter [28]. For measurement of the promoter activity of protein-coding genes, test promoter sequences upstream from the corresponding translation initiation codons up to about 500 bp were PCR-amplified and inserted into pGRP between *Bgl*II and *Eco*T22I sites (the sites for *Bgl*II and *Eco*T22I were included in the PCR primers) [28]. For measurement of the activity of rRNA promoters without SD sequence, we constructed pGRS vector by adding, into pGRP vector, a 33 bp-long DNA fragment including the SD (AGGAGG) signal-containing sequence [AGATCT(*Bgl*II)TCGGAATTC(*Eco*RI)AAACAGGAGG(SD)ATTACCCCATGCAT(*Eco*T221)] between *Bgl*II and *Eco*T221 sites. About 500 bp-long sequence of seven *rrn* operons, each including both P1 and P2 promoters, were inserted into this pGRS vector in front of this synthetic SD sequence to construct the rRNA promoter assay vectors (pGRS-rrnA to pGRS-rrnH) (Table 1; see S1 Table for primer sequences for pGRS construction and Fig 4 for the plasmid map).

### Promoter assay

The promoter activity was determined by measuring GFP and RFP fluorescence according to the standard procedure [28]. In brief, the promoter assay vectors (pGRS-rrnA to pGRS-rrnH) constructed in this way (see Table 1) were transformed into the appropriate host strains. LB contains unidentified natural substances that are fluorescent that bothered the accurate measurement of fluorescence. For accurate measurement of the fluorescence intensity of RFP or GFP expressed in *E*. *coli*, cells grown in LB for various times were harvested by centrifugation, resuspended in phosphate-buffered saline, and diluted with phosphate-buffered saline to obtain approximately the same cell density (0.6 A600nm unit) for all samples. For measurement of bulk fluorescence, aliquots of a 0.3-mL cell suspension were added to 0.4 x 96 flat-bottom wells, and the fluorescence was measured with a FL600 Bio-Tek Microplate Fluorescence Reader (Bio-Tek Instruments, Winooski). The net fluorescence value was measured after subtraction of the background fluorescence, which was determined by using *E*. *coli* cultures with pGRS vector without promoter insertion.

### Purification of Fis protein

Fis protein was expressed using pFis expression vector that was constructed using pET21a vector, and affinity-purified using the standard purification procedure of His-tagged transcription factors [63]. The purity of His-tagged Fis was more than 95% as judged by PAGE.

### Gel shift assay

Promoter probes carrying seven rRNA promoter regions were generated by PCR amplification using pGRS reporter plasmids as template, Ex *Taq* DNA polymerase (Takara, Japan), and pairs of *rrn* primers (for primer sequences see S1 Table), one of which was labeled with 5’-fluorescein isothiocyanate [FITC], FITC-labeled PCR products were purified by PAGE prior to gel shift assays. Gel shift assays were performed under the standard conditions as described [64].

## Supporting Information

S1 Fig [A] Difference of 16S rRNA gene between seven *rrn* operons in *E*. *coli* K-12 W3110. The sequence of 16S rRNA gene was compared between seven *rrn* operons. The position that is different from the consensus sequence is shown in gray. The level of difference within a total of 1,542 bases of 16S rRNA is indicated in the difference column. [B] Difference of 23S rRNA gene between seven *rrn* operons in *E*. *coli* K-12 W3110. The sequence of 23S rRNA gene was compared between seven *rrn* operons. The position that is different from the consensus sequence is shown in gray. Extra two bases at positions 1883 and 1884 exist only in the *rrnA* operon, and thus these positions are shown in black for other six *rrn* operons. The level of difference within a total of 1,542 bases of 16S rRNA is indicated in the difference column.
(PDF)

S1 Table Primers used for isolation of rRNA promoters. For cloning the promoter region sequence upstream from 16S rRNA gene of each *rrn* operon, 313 to 554 bp sequences, shown in Fig 2, were PCR-amplified using the indicated primer sets.
(PDF)

## Abbreviations

RNAP: RNA polymerase
SELEX: systematic evolution of ligands by exponential enrichment
TF: transcription factor
GFP: green fluorescent protein
RFP: red fluorescent protein
