# Supplementary material for: Strength and Regulation of Seven rRNA Promoters in Escherichia coli
Source: PLoS One. 2015 Dec 30;10(12):e0144697. doi: 10.1371/journal.pone.0144697 (PMC4696680; doi:10.1371/journal.pone.0144697)
Supplement: S1 Fig — The sequence of 23S rRNA gene was compared between seven rrn operons. The position that is different from the consensus sequence is shown in gray. Extra two bases at positions 1883 and 1884 exist only in the rrnA operon, and thus these positions are shown in black for other six rrn operons. The level of difference within a total of 1,542 bases of 16S rRNA is indicated in the difference column. (PDF) [file pone.0144697.s001.pdf]

S1A Fig

Difference of 16S rRNA gene between 7 *rrn* operons

	Position in 16S rRNA																							
	5' domain													3' domain										
Gene	79	80	89	90	93	131	182	204	208	226	250	252	272	1002	1006	1010	1019	1020	1022	1023	1038	1120	Difference	
Helix	6					7	9	10		7	11			33								39		
rrsA																							1	0.07%
rrsB																							0	0
rrsC																							6	0.39%
rrsD																							0	0
rrsE																							9	0.58%
rrsG																							8	0.52%
rrsH																							9	0.58%
	Total number of nucleotides of 16S rRNA = 1,542																						33	0.31%

S1B Fig

Difference of 23S rRNA gene between 7 *rrn* operons

	Position in 23S rRNA																		
Gene	264	285	316	356	357	542	846	1171	1174	1175	1176	1177	1178	1179	1181	1182	1184	1187	1188
Helix	14	21	19	18		25	35	45											
rrlA																			
rrlB																			
rrlC																			
rrlD																			
rrlE																			
rrlG																			
rrlH																			

	Position in 23S rRNA																		
Gene	1211	1220	1229	1723	1725	1726	1727	1730	1733	1734	1735	1865	1869	1870	1871	1873	1874	1876	1877
Helix	46			63								68							
rrlA																			
rrlB																			
rrlC																			
rrlD																			
rrlE																			
rrlG																			
rrlH																			

	Position in 23S rRNA															
Gene	1878	1879	1880	1883	1884	2133	2203	2211	2256	2525	2794	2796	2800	2802		
Helix	68					78	79		80	91	94			98	Difference	
rrlA															**26**	**0.89%**
rrlB															**12**	**0.41%**
rrlC															**7**	**0.24%**
rrlD															**5**	**0.17%**
rrlE															**9**	**0.31%**
rrlG															**12**	**0.41%**
rrlH															**6**	**0.21%**
	Total number of nucleotides of 23S rRNA = 2,904														**77**	**0.38%**
