# Supplementary material for: Strength and Regulation of Seven rRNA Promoters in Escherichia coli
Source: PLoS One. 2015 Dec 30;10(12):e0144697. doi: 10.1371/journal.pone.0144697 (PMC4696680; doi:10.1371/journal.pone.0144697)
Supplement: S1 Table — For cloning the promoter region sequence upstream from 16S rRNA gene of each rrn operon, 313 to 554 bp sequences, shown in Fig 2, were PCR-amplified using the indicated primer sets. (PDF) [file pone.0144697.s002.pdf]

S1 Table. Primers used for isolation of rRNA promoters

Primer	Sequence
rrnA forward	GAAGATCTCGTTATCGCTGGTACGACCG
rrnA reverse	GGAATTCAAAATGTTGACGCTCAAAGAATTAAACTT
rrnB forward	GAAGATCTGATTCTGGCGCAGCGATTGC
rrnB reverse	the same with *rrnA* reverse
rrnC forward	GAAGATCTGAAAGGTTTTTCTGTGCAGC
rrnC reverse	GGAATTCAAAAGTTTGATGCTCAAAGAATTAAACTTCG
rrnD forward	GAAGATCTCGATCATTACGCGCTGACCG
rrnD reverse	the same with *rrnC* reverse
rrnE forward	GAAGATCTCTCAGCTAACGCCCCTAACG
rrnE reverse	the same with *rrnC* reverse
rrnG forward	GAAGATCTGCAGCAGATCGAAAACCCGCTGG
rrnG reverse	the same with *rrnC* reverse
rrnH forward	GAAGATCTGATCTGCAGGCAGCGGTTGC
rrnH reverse	GGAATTCAAAAGTTTGATGCTCAAAGAATTAAACTTTG
lacUV5 forward	GAAGATCTCAGCTGGCACGACAGGTTTC
lacUV5 reverse	CGATGCATAGCTGTTTCCTGTGTGAAATTG
